# Altered retrieval of melodic information in congenital amusia: insights from dynamic causal modeling of MEG data

**DOI:** 10.3389/fnhum.2015.00020

**Published:** 2015-02-04

**Authors:** Philippe Albouy, Jérémie Mattout, Gaëtan Sanchez, Barbara Tillmann, Anne Caclin

**Affiliations:** ^1^Lyon Neuroscience Research Center, Auditory Cognition and Psychoacoustics Team, CRNL, CNRS UMR5292, INSERM U1028; University Lyon 1Lyon, France; ^2^Lyon Neuroscience Research Center, Brain Dynamics and Cognition Team, CRNL, CNRS UMR5292, INSERM U1028; University Lyon 1Lyon, France; ^3^Montreal Neurological Institute, McGill UniversityMontreal, QC, Canada

**Keywords:** tone deafness, effective connectivity, short-term memory, magneto-encephalography, pitch processing

## Abstract

Congenital amusia is a neuro-developmental disorder that primarily manifests as a difficulty in the perception and memory of pitch-based materials, including music. Recent findings have shown that the amusic brain exhibits altered functioning of a fronto-temporal network during pitch perception and short-term memory. Within this network, during the encoding of melodies, a decreased right backward frontal-to-temporal connectivity was reported in amusia, along with an abnormal connectivity within and between auditory cortices. The present study investigated whether connectivity patterns between these regions were affected during the short-term memory retrieval of melodies. Amusics and controls had to indicate whether sequences of six tones that were presented in pairs were the same or different. When melodies were different only one tone changed in the second melody. Brain responses to the changed tone in “Different” trials and to its equivalent (original) tone in “Same” trials were compared between groups using Dynamic Causal Modeling (DCM). DCM results confirmed that congenital amusia is characterized by an altered effective connectivity within and between the two auditory cortices during sound processing. Furthermore, right temporal-to-frontal message passing was altered in comparison to controls, with notably an increase in “Same” trials. An additional analysis in control participants emphasized that the detection of an unexpected event in the typically functioning brain is supported by right fronto-temporal connections. The results can be interpreted in a predictive coding framework as reflecting an abnormal prediction error sent by temporal auditory regions towards frontal areas in the amusic brain.

## Introduction

Congenital amusia refers to a neuro-developmental disorder characterized by impairments in pitch perception, production, and memory, more or less strongly accompanied by deficits along the time dimension (i.e., altered processing of rhythm or meter) (Ayotte et al., 2002; Peretz et al., 2002; Stewart, 2011; Peretz, 2013; Williamson and Stewart, 2013; Tillmann et al., 2015). The disorder cannot be explained by cognitive deficits, hearing loss, or brain damage (Ayotte et al., 2002; Peretz, 2013), and the pitch-related impairments seem to impact mostly the processing of musical material, with some consequences also in the speech realm (Ayotte et al., 2002; Peretz and Hyde, 2003; Patel et al., 2005, 2008; Liu et al., 2010). Although the seminal studies on congenital amusia have focused on impairments of pitch discrimination and direction judgments (Ayotte et al., 2002; Peretz et al., 2002; Peretz and Hyde, 2003; Foxton et al., 2004; Hyde and Peretz, 2004; Stewart et al., 2006; Jones et al., 2009; Stewart, 2011; Jiang et al., 2013), subsequent studies have suggested that this disorder could also be traced down to deficits in short-term memory for pitch (Gosselin et al., 2009; Tillmann et al., 2009, 2015; Williamson and Stewart, 2010; Williamson et al., 2010; Albouy et al., 2013a,b).

The pitch-specificity of the short-term memory deficit has been observed with numerous behavioral approaches. Data collected with a conventional “span” memory task with numbers (Williamson and Stewart, 2010), or with a delayed comparison task with one-syllable words (Tillmann et al., 2009) showed intact performance in congenital amusics for verbal material, while confirming impaired performance for musical material. Additionally, in delayed comparison tasks, amusic individuals’ performance is more strongly affected than control participants’ performance when the durations of the retention interval between single tones are increased (Gosselin et al., 2009; Williamson et al., 2010) and the length of the to-be compared tone sequences are increased (Gosselin et al., 2009). Finally, amusic individuals exhibit increased sensitivity (i.e., leading to decreased performance) to interference caused by irrelevant tones presented during the retention interval (Gosselin et al., 2009; Williamson et al., 2010).

The functional cerebral correlates of the pitch memory deficit in congenital amusia have been investigated more recently. Recording Magnetoencephalography (MEG) signals during a delayed comparison task with melodies, functional abnormalities were revealed during encoding, short-term retention, and short-term memory retrieval of the melodic information in amusic individuals as compared to the matched control participants who were typical non-musician listeners (Albouy et al., 2013a). During the encoding of melodies, the amusic brain elicited abnormal (decreased and delayed) N100m components in bilateral Inferior Frontal Gyri (IFG, pars opercularis) and auditory cortices. Abnormal functioning of fronto-temporal regions was also observed during other processing steps of the short-term memory task, as revealed by right-lateralized functional anomalies (in the Dorsolateral Prefrontal Cortex (DLPFC) and Posterior Parietal Cortex (PPC)) during the retention of pitch information, and by altered functioning of bilateral fronto-temporal (IFG, auditory cortex) regions during the short-term memory retrieval of melodies.

The functional abnormalities observed during a delayed comparison task with MEG recordings are in agreement with functional abnormalities observed during passive listening with fMRI (Hyde et al., 2011) and with anatomical abnormalities observed along the auditory-frontal pathway in the amusic brain (Hyde et al., 2006, 2007; Mandell et al., 2007; Loui et al., 2009). Overall, these findings were in agreement with data on the normal functioning (non-amusic) brain suggesting that pitch processing (perception and memory) involves both the auditory cortex and distant brain areas, notably frontal and parietal cortices (Griffiths, 1999; Griffiths et al., 1999; Maess et al., 2001; Janata et al., 2002a,b; Tillmann et al., 2003, 2006; Peretz and Zatorre, 2005; Koelsch et al., 2009; Foster and Zatorre, 2010; Schulze et al., 2011a,b; Schulze and Koelsch, 2012; Foster et al., 2013).

However, most of these studies described co-activation patterns of distant brain areas without analyzing the connectivity patterns within specific networks that are underlying participants’ task performance. While different cognitive processes may recruit similar brain regions, the connectivity patterns between these regions can differ depending on the context of the operations being actively performed (D’Esposito, 2007). Indeed, beyond functional specialization, the principle of functional integration in cognitive neuroscience suggests that complex cognitive processes are supported by dynamic interactions between different brain areas (Varela et al., 2001; Friston et al., 2003; Garrido et al., 2007; Rauschecker and Scott, 2009). Understanding these dynamical interactions is relevant not only in the normally functioning brain, but also in impairments and deficits, such as amusia. While the implication of anomalies in the fronto-temporal regions in amusics’ pitch deficits has now been reported in several studies, there remain open questions about whether and how altered connectivity patterns between these brain areas underlie the disorder.

Using functional connectivity measures on fMRI data during passive listening to pitch, Hyde et al. (2011) reported an increased lateral connectivity between the two auditory cortices and most importantly, a decreased connectivity between the right IFG and the right Superior Temporal Gyrus (STG) in the amusic brain in comparison to controls. This abnormal pattern of fronto-temporal connectivity has also been reported recently during resting state (Lévêque et al., submitted). Together with the anatomical abnormalities observed in this pathway with Diffusion Tensor Imaging (Loui et al., 2009), these findings led to the hypothesis that the cerebral correlates of congenital amusia could be related to decreased anatomical and functional fronto-temporal connectivity. However, although functional connectivity measures allow the establishment of statistical dependency between different brain regions, they do not provide information about the causal architecture of the interactions (Friston et al., 2003).

To improve the understanding of the effective connectivity patterns supporting auditory encoding of pitch information in the amusic brain, Albouy et al. (2013a) have used Dynamic Causal Modeling (DCM; David et al., 2006) of MEG data in a delayed comparison task. In this study, amusics’ altered encoding of auditory information (i.e., during the first melody of the delayed comparison task) was related to (1) reduced intrinsic connectivity within each of the auditory cortices; (2) increased lateral connectivity between right and left auditory cortices; and (3) a decreased right frontal-to-temporal (backward) connectivity in amusics relative to controls. These results suggest that abnormal causal interactions underlie the altered brain responses observed within the auditory fronto-temporal network in the amusic brain. Moreover, these results suggest that in the typical brain, the encoding of auditory information, which allows keeping a memory trace of a previously presented stimulus in order to compare it (top-down) to a sound occurring later (bottom-up), implies a crucial role of backward connections (from the right IFG to the right auditory cortex) to support the construction of an appropriate memory trace of the stimulus.

Along these lines, the present study aims at (1) improving the characterization of the cerebral correlates of short-term memory deficits in congenital amusia; and (2) extending our understanding of the mechanisms supporting auditory short-term memory in the typically functioning brain.

We here further analyzed the MEG data acquired in the study of Albouy et al. (2013a) where amusic participants and matched control participants performed a melodic contour task, in which two six-tone sequences had to be compared (same/different paradigm, also referred to as delayed comparison task). When the melodies were different, only one tone changed in the second melody. Focusing on this changed tone, Albouy et al. (2013a) have previously analyzed amplitude and source localization of Event Related Fields (ERFs) for the difference between “Different” and “Same” trials (for correct responses). While the control group showed activity in bilateral auditory cortices and in the pars opercularis of the IFG (BA 44) when they detect the changed tone, the amusic brain showed strongly reduced brain responses in these regions (see Albouy et al., 2013a).

Using data from the same experiment, we performed three types of DCM analyses investigating the ERFs during the second melody of the delayed comparison task: (1) By comparing amusics and controls for “Same” trials, we explored whether the previously reported anomalies in the amusic brain during the encoding of pitch (i.e., when processing the first melody of the pair in the delayed comparison task) are also present during short-term memory retrieval (i.e., when processing the second melody of the pair); (2) By comparing “Different” and “Same” trials for controls only, we investigated what kind of connectivity patterns support the detection of a deviant tone in the typically functioning brain; and (3) By comparing amusics and controls for the “Difference Wave” (“Different” minus “Same” trials), we investigated whether amusics’ altered brain responses related to the detection of the changed tone (short-term memory retrieval) could be also associated with abnormal connectivity patterns within this bilateral fronto-temporal network.

## Methods

In the present article, we performed new DCM analyses of the data from Albouy et al. (2013a). All details about the experiment (participants, materials, procedures, MEG pre-processing, and source reconstruction analyses) are described in *Material and Methods* of the original paper (from p. 1640–1646). We here present a short summary of the methods, and focus on the DCMs used to investigate the modulations of effective connectivity that support the short-term retrieval of pitch information.

### Participants

Nine amusic individuals and nine matched non-musician controls participated to the study. In a previous testing session, all participants were tested with the Montreal Battery of Evaluation of Amusia (Peretz et al., 2003) and with a two-alternative forced-choice task (using a staircase procedure) to evaluate their pitch discrimination thresholds (Tillmann et al., 2009). Participants’ demographic characteristics and data from pre-tests are presented in Table 1. Ethical approval was obtained from the French ethics committee on Human Research (CPP Sud-Est II, #2006-018/A-1).

**Table 1:** **Demographic characteristics of participants and their data for behavioral pretests**.

Characteristics	Amusics (*n* = 9)	Controls (*n* = 9)	*t*-test
*Age in years*	31.5 (8.5)	31.33 (7.3)	*NS*
*Gender*	5 females, 4 males	5 females, 4 males	
*Education in years*	14.8 (1.7)	16.1 (2.6)	*NS*
*Musical education in years*	1.2 (1.9)	0.7 (1.2)	*NS*
MBEA Peretz et al. (2003)			
*Mean score (cut off score = 23.4)*	20.9 (1.7)	27.6 (0.8)	*t*_(16)_ = 10.6, *p* < 0.001
*Melodic sub-tests (cut off score = 21.6)*	19.8 (2.6)	27.9 (1.6)	*t*_(16)_ = 9.75, *p* < 0.001
Pitch discrimination threshold Tillmann et al. (2009)
*Threshold in semitones*	1.07 (1.2)	0.3 (0.3)	*t*_(16)_ = 1.83, *p* = 0.04

*Educational background is calculated in years of education starting from the first year of primary school in the French system, at about 6 years of age. Results of the Montreal Battery of Evaluation of Amusia (MBEA) are expressed as number of correct responses (average over the six sub-tests of the battery, maximum score = 30; and average over the three melodic subtests, maximum score = 30). Pitch Discrimination Threshold (PDT) scores are reported in semitones. Data are reported as a function of group, along with significance levels on corresponding *t*-tests; “NS” refers to a non-significant difference (*p* > 0.05). Standard deviations are in parentheses*.

### Delayed comparison task and procedure

**Figure 1:**
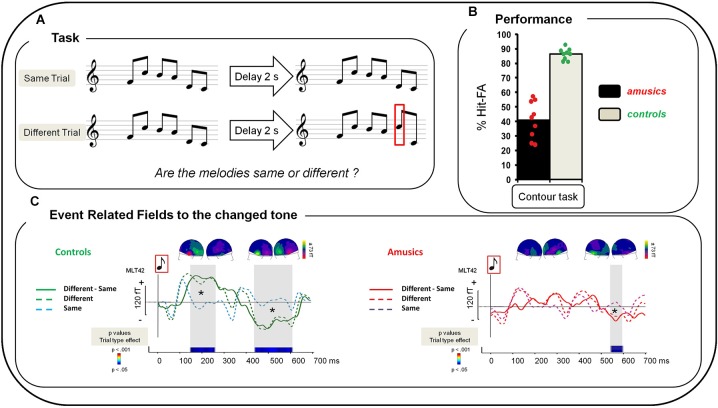
**(A)** Examples of the musical stimuli. “Same” trials: After a 2 s delay, S1 was repeated as the second melody of the pair (S2). “Different” trials: one tone was changed in the second melody of the pair (red square). **(B)** Performance of amusic and control groups (Gray, Controls; Black, Amusics) in terms of percent of Hit-FA. Green circles: controls’ individual performance; red circles: amusics’ individual performance. **(C)** Grand average of a left temporal MEG sensor (MLT42) for a 0–700 ms time window after the onset of the changed tone in S2 for the Contour Task for each group and each type of trial. Left: For controls. Green dotted line: “Different” trials, correct responses; blue dotted line: “Same” trials, correct responses; green plain line: “Difference Wave” (“Different” trials—“Same” trials for correct responses). Right: For amusics. Red dotted line: “Different” Trials, correct responses; purple dotted line: “Same” trials, correct responses; red plain line: “Difference Wave” (“Different” trials—“Same” trials for correct responses). Sensor plots correspond to the mean event-related fields (ERFs) of the “Difference Wave” in the 150–250 ms and the 400–600 ms time-windows for the change tone (average of all participants of each group).Two-sample *t*-tests were performed at each time sample on sensor amplitudes in the 0–700 time window in the two groups of participants. *p*-values are reported across time in the lower panel with blue for *p* < 0.05; green for *p* < 0.01; and red for *p* < 0.001. Note that only effects lasting longer than 15 ms were reported. See Albouy et al. (2013a) for details.

Participants performed a delayed comparison task for which they had to compare two six-tone sequences (S1, S2) separated by a silent retention period of 2000 ms. The two tone sequences could be either the same or different. All sequences were composed of six 250-ms piano tones presented successively without inter-stimulus-interval. One hundred and ninety two different melodies (sequences) were created using eight piano tones differing in pitch height (Cubase software, Steinberg); all used tones belonged to the key of C Major (C3, D3, E3, F3, G3, A3, B3, C4). These 192 sequences were used as S1. For S2 in “Different” trials, one tone (in positions 2–5) was replaced by a different tone of the set to create a contour-violation in the melody (Figure 1A). Participants performed the melodic task and another control task (not presented here, see Albouy et al., 2013a) during the MEG recording. Presentation software (Neurobehavioral systems, Albany, CA, USA) was used to run the experiment and to record button presses. For each trial, participants had to decide whether S2 was identical to S1 or different from S1. There were six blocks separated by 2–3 min of break. Note that there were also six blocks of the control task that were presented in alternation (counterbalanced across participants). Participants were informed of task order and asked to indicate their answers by pressing one of two keys with their right hand after the end of S2. They had 2 s to respond before the next trial occurring 2.5 s–3 s after the end of S2. No feedback was given during the experiment. In each block, 32 trials were presented (16 same pairs, 16 different pairs), resulting in 192 trials in total.

### MEG recordings and analyses

The recordings were carried out using a 275-channel whole-head MEG system (CTF-275 by VSM Medtech Inc., Vancouver, Canada) with continuous sampling at a rate of 600 Hz, a 0–150 Hz filter bandwidth, and first-order spatial gradient noise cancellation. Horizontal and vertical electrooculograms (EOG) and electrocardiogram were acquired with bipolar montages. Head position was determined with coils fixated at the nasion and the preauricular points (fiducial points, continuous sampling at a rate of 150 Hz). Participants were seated upright in a sound-attenuated, magnetically-shielded recording room, and listened to the sounds presented binaurally through air-conducting tubes with foam ear tips. Prior to the MEG recording, participants’ sound detection thresholds (using G3, the tone in the center of the tone set for S1) were determined for each ear, and the level was adjusted so that the sounds were presented at about 50–55 dB Sensation Level with a central position (stereo) with respect to the participant’s head. MEG data were first analyzed in sensor space using CTF tools (VSM Medtech Inc., Vancouver, Canada) and the ELAN software package developed in the Brain Dynamics and Cognition team (Lyon Neuroscience Research Center^1^; Aguera et al., 2011). Source reconstruction and DCM analyses were performed with SPM8 (Wellcome Trust Centre for Neuroimaging, London, UK; Litvak et al., 2011) using MATLAB 7.6 (Mathworks Inc., Natick, MA, USA).

Individual MEG trials were automatically inspected from −100 ms to 5500 ms with respect to the onset of the first S1 tone (i.e., a time window covering S1, the delay, S2 and an additional 500 ms after S2). Trials with ranges of values exceeding ± 3000 fT within a 1000 ms sliding time-window at any sensor site (±100 μV at EOG channels) were excluded from the analysis: as a result, between 90 and 165 trials were kept for each participant and condition. After artifact rejection, two different second-order Butterworth filters (12 dB/octave slope) were performed: (1) for transient evoked responses, a band-pass filter between 2 and 30 Hz; and (2) for the analysis of the change-specific responses, filtering was done with a band-pass filter between 0.5 and 30 Hz.

The analyses reported here focused on ERFs evoked by (1) the short-term memory retrieval of “Same” trials, corresponding to the average of tones 2–6 of the second melody of the pair (see procedure and rationale in Albouy et al., 2013a); (2) the changed tone in S2 for correct responses (i.e., during the detection of the changed tone) (Figure 1C). Note that because control participants had more correct responses than amusic participants, we used for each control participant the same number of trials as his/her matched amusic participant (selected randomly from the entire set of correct response trials). To analyze event-related responses following the “changed” tone in S2, two averages were performed for each participant (note that we always kept the baseline in the −100 to 0 ms interval before S1): firstly, an average of all correctly detected changed tones (in position 2–5), in a −100 to 700 ms time-window around the onset of the change (this ERF thus combined data for differences in all possible positions in S2), and secondly, an average of tones from correctly classified “Same” trials with, for each participant, the same number of tones in position 2, 3, 4, or 5 as used for the ERFs of the “Different” trials.

For controls only, the change-specific response was also assessed by comparing “Different” and “Same” trials (using the equivalent (unchanged) tone (in the same position of the melody as the changed tone of the “Different” trial)) with DCM (for correct responses). As DCM attempts to explain differences between waveforms in terms of coupling changes among sources, we considered that, in amusics, the waveforms for “Same” and “Different” trials were too similar (see Figure 1C) to allow comparing them with a DCM analysis.

### Dynamic causal modeling

We used DCM as implemented in SPM8 (David and Friston, 2003; David et al., 2006; Garrido et al., 2007; Litvak et al., 2011). DCM uses the concept of effective connectivity, which refers explicitly to the influence that one neuronal system exerts over another. DCM models interactions among cortical regions and allows making inferences about system parameters and the influence of experimental factors on these parameters. This analysis method uses neural mass models (David and Friston, 2003) to explain source activity in terms of the ensemble dynamics of interacting inhibitory and excitatory subpopulations of neurons (Jansen and Rit, 1995). It emulates the activity of a cortical source using three neural subpopulations, each assigned to one of three cortical layers: (1) an excitatory subpopulation in the granular layer; (2) an inhibitory subpopulation in the supra-granular layer; and (3) a population of deep pyramidal cells in the infra-granular layer. In this model, the excitatory pyramidal cells receive excitatory and inhibitory inputs from local inter-neurons (via intrinsic connections, confined to the cortical sheet), and send excitatory outputs to remote cortical areas via extrinsic connections. Bottom-up connections (also referred to as forward connections) originate in the infra-granular layers and terminate in the granular layer. In contrast, top-down connections (also referred to as backward connections) link agranular layers and lateral connections originate in infra-granular layers and target all layers. Additionally, the model considers that all extrinsic cortico-cortical connections are excitatory and are mediated through the axons of pyramidal cells. Exogenous inputs to the model have the same characteristics as forward connections. By adopting this network architecture, DCM is able to assess how a given experimental manipulation activates a cortical pathway rather than a cortical area or source. This approach thus uses a biologically informed model that allows for inferences about the underlying neuronal networks generating evoked responses such as Event Related Potentials (ERPs) and ERFs.

Three different DCM analyses were performed: (1) the first analysis aimed at testing whether amusics’ altered brain responses during short-term memory retrieval of “Same” trials (without changed tone) could be explained by changes in effective connectivity between bilateral auditory cortices and bilateral IFG, as well as within and between the two auditory cortices; (2) the second analysis investigated what kind of connectivity patterns support the detection of a deviant tone in the typically functioning brain. We compared and modeled in controls (grand average data of the controls) the difference in effective connectivity between “Different” and “Same” trials (for correct responses); (3) the third analysis aimed at testing whether amusics’ altered brain responses during the detection of the changed tone (“Difference Wave”) could be explained by changes in effective connectivity between sources of the same network. We aimed at characterizing the two participant groups with a high signal-to-noise ratio and to investigate the putative differences in effective connectivity between them. DCM analyses were thus applied to the grand average data at the sensor level. This is equivalent to a meta-subject analysis (corresponding to the average of nine participants per group). It is relevant to note that this approach comes with an anatomo-functional approximation in the sense that it disregards the inter-subject variability within each group. However, this approach makes it possible to test, with DCM, alternative mechanistic hypothesis about the network architecture and modulations in effective connectivity that differ (1) between amusic and control participants (analyses 2 and 3); and (2) between two conditions within a group (analysis 2). At the expense of assuming a meta-subject for each group, this approach provides quantitative conclusions at the population level that benefit from a higher signal-to-noise ratio and hence a greater sensitivity. We thus compared and modeled the difference between the grand average data of the controls (average over the nine participants) and the grand average data of the amusics (average over the nine participants at the sensor level) (as in Albouy et al., 2013a) and the model comparisons were thus based on Fixed effects.

**Figure 2:**
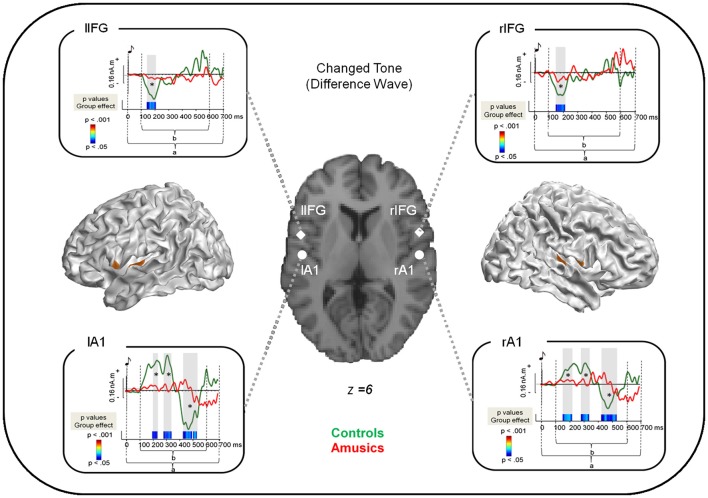
**Source reconstruction of the brain responses specifically evoked by the changed tone in S2**. Cortical meshes show bilateral regions that were significantly different from baseline (as indicated by the brown areas). Coordinates of the peaks of activations are displayed on the single subject T1 image provided by SPM8 for four regions: the bilateral auditory cortices as well as the bilateral pars opercularis of the Inferior Frontal Gyrus (see Table 2). The surrounding panels correspond to the grand average of source data for each region and for the time window where the inversion was performed (0–700 ms after the changed tone onset, as indicated by a) for the control group (green) and the amusic group (red). Two sample *t*-tests were performed at each time sample and for each region on source amplitude in the 100–600 ms time window (as indicated by b) in the two groups of participants. *p*-values are reported across time below the source amplitudes with blue for *p* < 0.05; green for *p* < 0.01; and red for *p* < 0.001. Note that only effects lasting longer than 15 ms were reported. See Albouy et al., 2013a for details.

For analysis (1) we modeled the data during the post-stimulus period 0–250 ms (corresponding to the inter-tone interval in melodies (see Albouy et al., 2013a). For analyses (2) and (3), we modeled the data during the post-stimulus period 0–700 ms. This latter period encompasses components of the ERFs that are assumed to reflect the detection of the changed tone (see Figures 1C, 2). Indeed, as described in Albouy et al. (2013a) and below, the processing of the changed tone in controls was associated with two evoked responses. The first evoked response was elicited approximately 150 ms after the onset of the changed tone; and the second one peaked at 500 ms after the onset of the changed tone.

**Table 2:** **Frontal and temporal generators of the change-specific response within S2**.

Lobe	Region	Hemisphere	*x*	*y*	*z*	mm^2^	nb
Frontal	IFG, opercular part	R	55	4	6	49	10
		L	−54	3	6	65	12
Temporal	STG/PT	R	55	−12	5	182	25
		L	−52	−12	5	117	16

*Coordinates correspond to the vertex with maximal amplitude within each region (coordinates are in MNI space). IFG, Inferior Frontal Gyrus; STG, Superior Temporal Gyrus; PT, Planum Temporale; nb, number of vertices*.

### Network model specification and bayesian model selection

**Figure 3:**
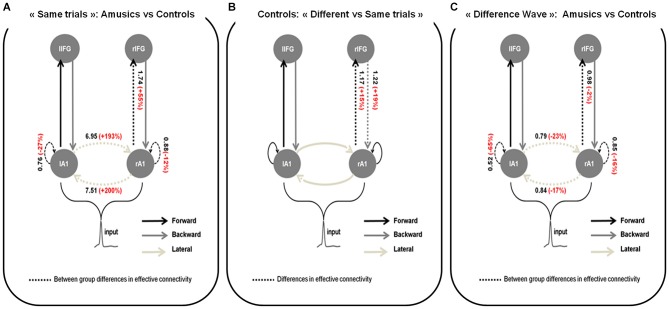
**Wining models for **(A)** Amusics vs. Controls comparison for “Same” trials; **(B)** “Different” vs. “Same” trials in S2 for controls and; **(C)** Amusics vs. Controls for the “Difference Wave”**. Dashed arrows indicate modulated connections (i.e., connections that differ between groups **(A,C)** or type of trial **(B)**) and solid arrows indicate fixed connections. Significant changes in effective coupling are specified (in black: amount of coupling change between groups **(A,C)** or type of trials **(B)**; in red: corresponding relative coupling with amusics coupling expressed in % of control coupling **(A,C)** or with coupling for “Different” trials expressed in % of coupling for “Same” trials **(B)**).

All compared models were based on the same network architecture, which was motivated by (1) the results of our classical source reconstruction analysis of the brain responses evoked by the changed tone, which were revealing sources in a bilateral fronto-temporal network (see Table 2 and Albouy et al., 2013a for ROIs that were significantly different from baseline); and (2) the hypothesis of impaired fronto-temporal connectivity and inter-hemispheric connectivity, which were observed in congenital amusia with functional (Hyde et al., 2011), effective (Albouy et al., 2013a) and anatomical connectivity approaches (Loui et al., 2009). We assumed four sources, modeled as equivalent current dipoles (ECDs), over left and right primary auditory cortices (A1), left and right pars opercularis of the IFG (see Table 2 and Figure 2). Using these sources, we constructed the following DCM (as in Albouy et al., 2013a): An extrinsic input entered bilaterally to the primary auditory cortices (A1), which were connected to their ipsilateral IFG. Inter-hemispheric (lateral) connections were placed between left and right A1. All connections were reciprocal (i.e., connected with forward and backward connections or with bilateral connections, see Figure 3). Given this network architecture, we used a (four) factorial design and performed family-level inference to assess modulations of effective connectivity underlying (1) the group difference in auditory evoked responses (analyses 1 and 3); and (2) the differences between “Different” and “Same” trials in controls (analysis 2). In all analyses, the first Factor pertains to the modulation of intrinsic connectivity in bilateral auditory cortices (and includes two families (or levels), corresponding to models where these intrinsic connections were modulated (or not) between the two groups/conditions). The second Factor pertains to the modulation of lateral connections between the two auditory cortices (two families: models that include a modulation or not). The third Factor relates to the type of connections between auditory and frontal areas that are modulated (that is, forward, backward, or both forward and backward connections) or not (resulting in four families). Finally, the fourth Factor pertains to the hemispheric location of the above described modulated connections, either in the right hemisphere, the left hemisphere, or both (resulting in three families). We thus fitted and compared 48 models for each analysis. Assuming uniform prior probabilities over families, we used Bayesian model selection (BMS) to compare them in fixed effects analysis (FFX; Penny et al., 2010). This rests upon the free energy (or approximate marginal likelihood or evidence) for each model, and yields a posterior probability associated with each model family.

## Results

Behavioral and MEG results of the present experiment were reported in detail in Albouy et al. (2013a; p. 1646–1655). This result section presents a short summary of the behavioral and MEG results to support the comprehension of the new DCM analyses performed on the data.

### Behavioral data

Performance was significantly above chance (i.e., 0% of Hits—False Alarms (FAs)) in each group (*t*-tests, all *ps* < 0.0001). Hits-FAs were analyzed with a one-way ANOVA and revealed that the main effect of Group (*F*_(1,16)_ = 103.83; *p* < 0.0001; *MSE* = 88.39; $\eta_{p}^{2}$ = 0.86), was significant: all amusic participants exhibited a deficit in the melodic contour task in comparison to controls (Figure 1B).

To investigate participants’ overall strategy (missing pitch changes or hearing non-existing changes), additional analyses were performed by investigating the effect of Trial Type (same, different) in participants’ performance. We analyzed the percentage of correct responses with a 2 × 2 ANOVA, with Group as between-participants factor and Trial Type (same, different) as within-participant factors. In addition to the main effect of Group (*F*_(1,16)_ = 107.06; *p* < 0.0001; *MSE* = 43.0; $\eta_{p}^{2}$ = 0.86), the effect of Trial Type was significant (*F*_(1,16)_ = 49.97; *p* < 0.0001; *MSE* = 73.4; $\eta_{p}^{2}$ = 0.75) revealing that participants’ performance was decreased for “Different” trials in comparison to “Same” trials. Finally, the Group-by-Trial-Type interaction was significant (*F*_(1,16)_= 28.37; *p* < 0.0001; *MSE* = 73.4; $\eta_{p}^{2}$ = 0.63). To analyze this interaction, Fischer LSD *post hoc* tests were carried out and revealed that while controls show similar performance for “Same” and “Different” trials (*p* = 0.23) amusics show decreased performance for “Different” trials as compared to “Same” trials (*p* < 0.001). Note that amusic performance was decreased as compared to controls for both “Same” (*p* = 0.048) and “Different” trials (*p* < 0.0001).

### MEG results

#### Source analyses of the transient responses of the changed tone in S2

We summarize here the analyses presented in Albouy et al. (2013a) concerning the brain responses evoked by the changed tone in the S2 melody of “Different” trials (for correct responses). The “Difference Wave” (“Different” trials minus “Same” trials) observed at the sensor level revealed that for controls, the processing of the changed tone was associated with two evoked responses: The first one was elicited approximately 150 ms after the onset of the changed tone; and the second one peaked at 500 ms after the tone onset. This biphasic response was altered in amusics (Figure 1C, right panel).

Source modeling of the “Difference Wave” (i.e., between “Different” trials and “Same” trials for correct responses, see Section Methods) revealed that activity was significantly different from baseline in bilateral fronto-temporal regions (see Table 2).

Two-sample *t*-tests (corrected for multiple comparisons, see Albouy et al., 2013a) revealed group differences in source amplitude, with higher amplitudes for control than for amusic participants in the four regions of interest and in the following time windows: (1) Right STG, 150–210 ms; 290–340 ms; 425–520 ms; (2) rIFG, 160–210 ms; (3) Left IFG (lIFG), 150–205 ms, and; (4) Left STG, 190–230 ms; 280–340 ms; 425–530 ms (Figure 2).

### Dynamic causal modeling

For each DCM analysis, the four family-wise inference allowed us to retained a winning model (see Figure 3) based on posterior probabilities (criterion of *p* > 0.99).

#### Group comparison for S2 in “Same” trials

Figure 3A shows the network architecture of the winning model as well as the conditional estimates of the connection strengths associated with the connections that were significantly modulated to explain the amusic response compared to the control response for S2 in “Same” trials. Posterior estimates obtained with the winning model enabled us to conclude that for S2 in “Same” trials, compared to controls, amusic participants showed an abnormally increased lateral connectivity between the two auditory cortices, decreased intrinsic modulations in both auditory cortices, and increased forward connectivity from the right auditory cortex to the right IFG.

#### Comparison between “Different” and “Same” trials for controls

In control participants (Figure 3B), increased right lateralized temporo-frontal connections (both Forward and Backward) best explain the brain responses to “Different” trials as compared to “Same” trials. We found strong evidence in favor of this lateralized network compared with the set of alternative hypotheses, with a high posterior model probability >0.99.

#### Group comparison for the “Difference Wave”

When investigating the “Difference Wave”, posterior estimates enabled us to conclude that, amusic participants, in comparison to controls, showed decreased lateral connectivity between the two auditory cortices, decreased intrinsic modulations in both auditory cortices, and decreased forward connectivity between the right auditory cortex and the right IFG (see Figure 3C).

#### Lateralization of the effects

Finally, to investigate the putative imbalance of the observed effects, between the right and left hemispheres, we investigated whether group differences reported above in terms of relative coupling (with amusics coupling expressed in % of control coupling) differed between the right and left auditory cortices. Therefore we compared with DCM (for the wining models of analyses 1 and 3) each amusic participant to his/her matched control participant. When comparing the modulation values of intrinsic connections with a paired *t*-test, between the right and left auditory cortices for “Same” trials in S2 (analysis 1) and for the “Difference Wave” (analysis 3), no significant difference was observed (all *ps* > 0.19).

## Discussion

The present study aimed at extending the characterization of cerebral correlates of the short-term memory deficit for pitch in congenital amusia. The data revealed that in addition to the functional abnormalities observed in the right fronto-temporal pathway during encoding of pitch information (Albouy et al., 2013a), amusics’ pitch deficits are also associated with an altered functioning of the same network (bilateral IFG and auditory cortex) during the short-term memory retrieval of melodies. To explain these group differences in terms of differences of coupling among sources, we performed group comparisons with DCM. These analyses revealed that in comparison to controls, the amusic brain is characterized by abnormal connections between and within the two auditory cortices as well as abnormal right forward temporal-to-frontal effective connectivity during short-term memory retrieval. These findings, along with additional analyses in controls, suggest that the short-term memory retrieval of melodic information and the detection of a deviant/unexpected tone in the normally functioning brain (leading to a prediction error, (Bastos et al., 2012; Dietz et al., 2014; Friston et al., 2014)) are supported by temporo-frontal interactions.

### Altered short-term retrieval of melodic information in congenital amusia

As reported in Albouy et al. (2013a), source reconstruction of the ERFs elicited by the changed tone (the “Difference Wave” between correct “Different” trials and “Same” trials) allowed us to observe activity in bilateral auditory cortices and bilateral pars opercularis of the IFG (BA 44) in the control group. This is in line with previous research suggesting that the short-term memory retrieval of pitch information involves (1) brain areas that support the perceptual representation of that information (Owen, 2000; D’Esposito, 2007; Grimault et al., 2014), and (2) areas of the Ventro-lateral Prefrontal Cortex (IFG), that has been described as supporting low-level mnemonic processes, such as rehearsal and retrieval in short-term recognition (Owen, 2000). In contrast, the amusic brain exhibited an abnormal recruitment of these regions for pitch retrieval. These data thus further reflect the functional correlates of the short-term memory deficit in congenital amusia (see Albouy et al., 2013a).

Most interestingly, while encoding and short-term memory retrieval of melodic information recruit similar brain regions, the present data showed that the connectivity patterns between these regions can differ between those two stages.

### Altered intrinsic and lateral connectivity in the auditory cortex of the amusic brain

During the short-term memory retrieval of pitch information, for both “Same” trials and for the “Difference Wave”, the amusic brain showed abnormally reduced intrinsic connections within the bilateral auditory cortices in comparison to controls. This finding supports the fact that while amusics’ auditory cortex seems to present near normal functioning in response to pitch information in a simple context (passive or active listening without memory) (Peretz et al., 2005, 2009; Moreau et al., 2009, 2013; Hyde et al., 2011; Peretz, 2013), it elicits abnormal responses when higher-level processing is required (Albouy et al., 2013a). Interestingly, reduced intrinsic connectivity within both auditory cortices in the amusic brain has been observed also during the encoding of pitch information (see Albouy et al., 2013a), thus suggesting that primary sensory areas are recruited during both encoding and retrieval in a short-term memory task (Zatorre et al., 1994; Griffiths, 1999; Griffiths et al., 1999; Owen, 2000; Curtis and D’Esposito, 2003; Gaab et al., 2003; Peretz and Zatorre, 2005; D’Esposito, 2007; Logie and D’Esposito, 2007; Schulze et al., 2009). Moreover, this hypothesis is in line with studies suggesting that the detection of deviant tones (Garrido et al., 2007, 2008, 2009a,b; Lieder et al., 2013) in an oddball paradigm (Mismatch Negativity, MMN; Näätänen, 1992) is also supported by intrinsic connections within the auditory cortex, as revealed by DCM analyses. However, it should be noted that in the present study, the comparison between “Different” and “Same” trials in controls, did not specifically show the implication of these intrinsic connections for the detection of pitch deviance. Based on the present data, we can only propose that modulations of intrinsic connections are reduced in congenital amusics in comparison to controls not only during encoding, but also during short-term memory retrieval of pitch information.

In addition to the altered functioning of auditory cortices during short-term memory retrieval of pitch information, the analysis of the “Difference Wave” revealed reduced lateral connection strengths between the right and left auditory cortices in amusics in comparison to controls. This result contrasts with the group comparison performed for S2 in “Same” trials (see above, Figure 3A) and with the abnormal connectivity pattern in the amusic brain reported for pitch perception, notably during both passive listening (Hyde et al., 2011) and active encoding in a short-term memory task (Albouy et al., 2013a). There, hyper-connectivity between the two auditory cortices has been observed in amusics as compared to controls. This abnormal hyper-connectivity between the two auditory cortices in the amusic brain might be a marker of the pitch processing deficit, as also observed in other developmental disorders (see Wolf et al., 2010) for dyslexia; and see (Hyde et al., 2011) for converging evidence in amusia) or rather reveal compensatory mechanisms of the amusic brain. This would suggest that amusics might compensate for an impoverished processing in the right auditory cortex by recruiting the contralateral auditory cortex. Note that for the group comparison performed on the “Difference Wave” (Figure 3C), we removed brain responses related to the encoding of the information of S2 (for which increased lateral connectivity is observed in amusics), by computing the difference between brain responses in “Different” trials and in “Same” trials. The ERFs studied here can thus be considered as being specific to the detection of the deviance in the “Different” trials (and thus, the difference between the information retrieved from memory and the presented tone). Results thus suggest that lateral connections have a role in detecting an auditory event that mismatches with a memory trace of a previously heard/memorized stimulus (here S1). However, note that the specific role of these lateral connections in pitch short-term memory retrieval was not demonstrated in the typically functioning brain (data of controls) in the present study (“Different” vs. “Same” trials in controls).

### Altered forward vs. backward connectivity between the rA1 and rIFG

The DCM analyses revealed abnormal connectivity in the amusic brain in a right fronto-temporal network during the short-term memory retrieval of melodic information (for both the processing of “Same” trials and the processing of the changed tone). This right-lateralized abnormality is in line with previous research showing functional and anatomical alteration of this pathway in congenital amusia (Loui et al., 2009; Hyde et al., 2011; Albouy et al., 2013a; Lévêque et al., submitted). Moreover, the present data further support the hypothesis that the processing of pitch information in the brain is, to some extent, asymmetric with a right-hemispheric predominance (with both hemispheres being involved) as it has been previously suggested based on musical deficits observed for patients with right-hemispheric brain lesions (Zatorre and Samson, 1991; Peretz, 1996, 2001; Peretz et al., 1997; Patel et al., 1998; Steinke et al., 2001; Nicholson et al., 2002, 2003; Stewart et al., 2006), and by neuroimaging studies in typical listeners (Zatorre et al., 1994, 2002; Griffiths, 1999; Griffiths et al., 1999; Janata et al., 2002a,b; Tillmann et al., 2003, 2006; Koelsch et al., 2005, 2009; Peretz and Zatorre, 2005; Hyde et al., 2008; Stewart et al., 2008; Schulze et al., 2009, 2011a,b).

However, while an abnormal frontal-to-temporal *backward* connectivity in the right hemisphere has been observed during the encoding of pitch information in the amusic brain (see Albouy et al., 2013a), the present analyses revealed that during short-term memory retrieval (for “Same” trials and the “Difference Wave”), amusics exhibited an abnormal temporal-to-frontal *forward* connectivity between the right auditory cortex and the right IFG in comparison to controls. According to Garrido et al. (2007), auditory evoked brain responses are mediated by interactions between fronto-temporal cortical areas. This differential role of forward and backward connections in auditory perception can be interpreted within the predictive coding framework (Bastos et al., 2012; Dietz et al., 2014; Friston et al., 2014).

According to predictive coding principles, neural systems are able to predict statistical regularities in the environment based on prior experience. In this view, neural systems can attenuate responses to predictable events (Lecaignard et al., submitted), thanks to top-down predictions (conveyed by backward connections (Friston, 2003)) and the minimization of deviations from these predictions (i.e., the minimization of prediction errors). In contrast, for unpredictable events, bottom-up prediction error signals (supported by forward connections (Penny et al., 2004)) emerge and report the “newsworthy” information from a lower hierarchical level (sensory input) that was not predicted by the higher level (prediction, memory trace of the prior experience). Along these lines, a prediction error can emerge due to either an inefficient construction of the prediction (no prediction or incorrect prediction) or to the occurrence of unpredictable events.

In the short-term memory paradigm used in the present study, it might be hypothesized that during short-term memory retrieval for “Same” trials, in the typically functioning brain, the neural system may minimize prediction error signals when the prior information (here S1) has been well encoded and the developed predictions (appropriate predictions) are fulfilled in the S2 melody (“Same” trials). In contrast, the perception of “Different” trials (that mismatches with predictions for one tone) may generate prediction errors signals. This hypothesis has received support from the DCM analysis performed in the control group (analysis 2), which showed increased forward effective connectivity between the right auditory cortex and the right IFG in “Different” trials as compared to “Same” trials. Moreover and interestingly, this increased temporo-frontal connectivity was associated with increased backward frontal-to-temporal connectivity that could be considered as an update of the prediction model after the reception of the prediction error signal.

In the amusic brain, however, it can be also hypothesized that disruptions in predictive coding can underlie the abnormal percepts. In Albouy et al. (2013a), altered backward connectivity during the encoding of melodic contour patterns in the amusic brain has been reported. According to the predictive coding principles described above, this result could be interpreted as follows: the neural system needs to keep a trace of a previously presented stimulus in memory (top-down processes) while processing a new stimulus arriving in the perceptual system (Demany and Semal, 2008). This is done in order to build an appropriate memory trace (and prediction) of the incoming melodic pattern. Observing decreased effective backward connectivity from the right IFG to the right auditory cortex in the amusic brain could be interpreted as reflecting a decreased or vague prediction (relying on top-down backward connections). By considering that the memory trace (as the basis for the prediction) has not been appropriately constructed (altered encoding), the amusic brain might have build incorrect prediction, and inappropriate prediction errors signals can thus emerge during short-term memory retrieval, and thus even for “Same” trials.

Interestingly, in the present study, we observed increased forward connectivity between the right auditory cortex and the right IFG in the amusic brain, as compared to controls, for the processing of S2 in “Same” trials. Along these lines it might thus be hypothesized that as the first melody of the pair has not been well encoded (see Albouy et al., 2013a), tones occurring in the perceptual system during short-term retrieval (S2) could be considered as deviant tones by the amusic brain. It should be considered however that these prediction errors signals should occur similarly for “Same” and “Different” trials as every event of S2 can be considered as unexpected (not or weakly predicted) by the amusic brain. This notably finds support in the group comparison for the “Difference Wave” (analysis 3) for which only a small difference in the forward connection (that conveys prediction error signal) was observed between amusics and controls (2%). This suggests that in both groups, the right auditory cortex is sending prediction error signals when the deviant tone occurs, but the mechanisms causing these signals might differ between the two groups (incorrect prediction in amusics, detection of unpredictable event in controls as shown by the comparison of “Same” and “Different” trials, analysis 2).

Overall, the present study showed that in the amusic brain, forward temporal-to-frontal connectivity seems to be altered during short-term memory retrieval of melodies, thus comforting the hypothesis of impaired functioning of the right fronto-temporal network in this developmental disorder (Loui et al., 2009; Hyde et al., 2011; Albouy et al., 2013a; Peretz, 2013; Tillmann et al., 2015). This result is of interest because it suggests that frontal and temporal cortices support both encoding and short-term memory retrieval of melodic information, but their interaction is modulated in different directions for the two processing steps related to memory.

## Conclusion

In congenital amusia, an altered functioning of a bilateral fronto-temporal network during the short-term memory retrieval of melodic information was associated with altered connectivity patterns in a right fronto-temporal network as well as within and between the two auditory cortices. These data improve our understanding about the role of frontal and temporal structures (including auditory cortices) and of the fronto-temporal pathway in music processing, as well as its impairment in this developmental disorder. To pursue the characterization of the deficits in congenital amusia, further research investigating these encoding and retrieval mechanisms for other types of auditory information (such as verbal material) as well as for other modalities (such as visual memory) are needed. This will allow determining whether connectivity patterns for visual memory (Sneve et al., 2013) are preserved or not in amusia, and if the altered brain responses and connectivity patterns observed in the amusic brain for tone sequences could be considered as a general impairment of auditory processing or are specific for pitch.

## Author contributions

Philippe Albouy, Barbara Tillmann and Anne Caclin designed the experiment, created the auditory stimuli, and programmed the tasks. Philippe Albouy, Barbara Tillmann, and Anne Caclin collected the data. Philippe Albouy analyzed the data. Jérémie Mattout and Gaëtan Sanchez provided the routines for the DCM analyses and supervised data analyses. Philippe Albouy, Jérémie Mattout, Gaëtan Sanchez, Barbara Tillmann and Anne Caclin interpreted the results and Philippe Albouy wrote the first draft of the article. All authors critically revised the article and approved the final version. The authors are accountable for all aspects of the work and ensure that questions related to the accuracy or integrity of any part of the work are appropriately investigated and resolved.

## Conflict of interest statement

The authors declare that the research was conducted in the absence of any commercial or financial relationships that could be construed as a potential conflict of interest.

## Footnotes

^1^http://elan.lyon.inserm.fr/
